# CENP-A Regulation and Cancer

**DOI:** 10.3389/fcell.2022.907120

**Published:** 2022-06-02

**Authors:** Charlène Renaud-Pageot, Jean-Pierre Quivy, Marina Lochhead, Geneviève Almouzni

**Affiliations:** Institut Curie, PSL Research University, CNRS, Sorbonne Université, Nuclear Dynamics Unit, Équipe Labellisée Ligue contre le Cancer, Paris, France

**Keywords:** CENP-A histone variant, centromere, cancer, chromosome instability, chromatin organization

## Abstract

In mammals, CENP-A, a histone H3 variant found in the centromeric chromatin, is critical for faithful chromosome segregation and genome integrity maintenance through cell divisions. Specifically, it has dual functions, enabling to define epigenetically the centromere position and providing the foundation for building up the kinetochore. Regulation of its dynamics of synthesis and deposition ensures to propagate proper centromeres on each chromosome across mitosis and meiosis. However, CENP-A overexpression is a feature identified in many cancers. Importantly, high levels of CENP-A lead to its mislocalization outside the centromere. Recent studies in mammals have begun to uncover how CENP-A overexpression can affect genome integrity, reprogram cell fate and impact 3D nuclear organization in cancer. Here, we summarize the mechanisms that orchestrate CENP-A regulation. Then we review how, beyond its centromeric function, CENP-A overexpression is linked to cancer state in mammalian cells, with a focus on the perturbations that ensue at the level of chromatin organization. Finally, we review the clinical interest for CENP-A in cancer treatment.

## Introduction

The first kinetochore proteins were described in 1985 using the sera of patients with an autoimmune disease called scleroderma (Moroi et al., 1980). William Earnshaw and Naomi Rothfield purified the autoantibodies targeting the centromeres of the patients and identified three proteins, including the Centromere Protein A: CENP-A (Earnshaw and Rothfield, 1985). Since then, biochemical approaches demonstrated that CENP-A is a variant of histone H3 (Palmer et al., 1987, 1991; Sullivan et al., 1994), which is one of the four major classes of histones (H2A, H2B, H3, H4) contributing to the basic unit of chromatin, the nucleosome. It is the only histone variant that is centromere-specific, its presence characterizing centromeric chromatin in most eukaryotes (Tromer et al., 2019). Within nucleosomes, CENP-A displays distinct features contributing to looser DNA binding and increased dynamics of the DNA ends in the nucleosome core particle (Sekulic et al., 2010; Panchenko et al., 2011; Tachiwana et al., 2011; Ali-Ahmad et al., 2019; Arimura et al., 2019). In addition, CENP-A-containing nucleosomes wrap only 121 bp of DNA, compared to the 147 bp in nucleosomes containing other H3 variants (Tachiwana et al., 2012; Hasson et al., 2013; Lacoste et al., 2014). These characteristics enable CENP-A to contribute to both the identity and function of centromeres. Centromeres are the loci where kinetochores assemble and dock to direct chromosome segregation during mitosis. Beyond this function during cell division, centromeres contribute to genome architecture as robust nuclear domains in interphase nuclei of mammalian cells (Dunleavy et al., 2009; Lacoste et al., 2014). Importantly, CENP-A has been involved as a key epigenetic determinant in defining the position of the centromere (Barnhart et al., 2011; Fachinetti et al., 2013). Furthermore, CENP-A is stably inherited across cell divisions, thereby helping to maintain centromere identity during cell proliferation (Jansen et al., 2007). Its inheritance through meiosis ensures a maintenance over organismal generations in mammals (Tyler-Smith et al., 1999; Amor et al., 2004; Smoak et al., 2016). CENP-A is also key for centromere function, establishing the interface necessary for the binding of components of the CCAN (constitutive centromere associated network) complex, such as CENP-C and CENP-N (Ali-Ahmad et al., 2019; Allu et al., 2019). At a chromatin level, CENP-A nucleosomes are interspersed with H3 nucleosomes along the arrays of repetitive DNA of the centromere (Dunleavy et al., 2011). Additional kinetochore components, CENP-T and CENP-W, associate with H3 nucleosomes (Hori et al., 2008). Both CENP-C and CENP-T interact with Mis12 and the Ndc80 complex, which are components of the KMN network linking the kinetochore to microtubules (Przewloka et al., 2011; Screpanti et al., 2011; Malvezzi et al., 2013). The level of CENP-A is thus key to maintain genome integrity, as shown by experimental alterations in CENP-A levels where both depletion (Maehara et al., 2010) and overexpression (Shrestha et al., 2017) promote mitotic defects and chromosomal instability (CIN) in human cells. Since alteration in CENP-A availability can alter both the formation of CENP-A-containing nucleosomes and centromere function, a link with cancer states is expected. Therefore, the control of CENP-A transcription, its turn-over/stability and its deposition into chromatin is central to ensure genome stability.

## Regulation of CENP-A

Histone chaperones escort non-nucleosomal (free) histones and ensure their deposition into chromatin (reviewed in (Ray-Gallet and Almouzni, 2021)). The timing of CENP-A deposition varies in different species. In mammals, the Holliday Junction Recognition Protein (HJURP) has been identified as the dedicated histone chaperone promoting CENP-A specific incorporation into centromeric chromatin, which in human occurs in late mitosis (outside S phase), to compensate for the replication-coupled dilution of CENP-A (Dunleavy et al., 2009; Foltz et al., 2009). Interestingly, in human cells, replication has been proposed as an error correction mechanism ensuring that CENP-A nucleosomes are retained at the centromere (Nechemia-Arbely et al., 2019). In addition, HJURP is involved in dealing with parental CENP-A displaced by the replication fork to ensure its recycling (Zasadzińska et al., 2018). This exclusive partnership is driven by the CENP-A targeting domain (CATD) in HJURP (Shuaib et al., 2010; Bassett et al., 2012). Furthermore, the presence of two C-terminal domains (HCTD1 and HCTD2) in HJURP is required for CENP-A deposition (Zasadzińska et al., 2013; Zhao et al., 2016; Pan et al., 2019).

**FIGURE 1:**
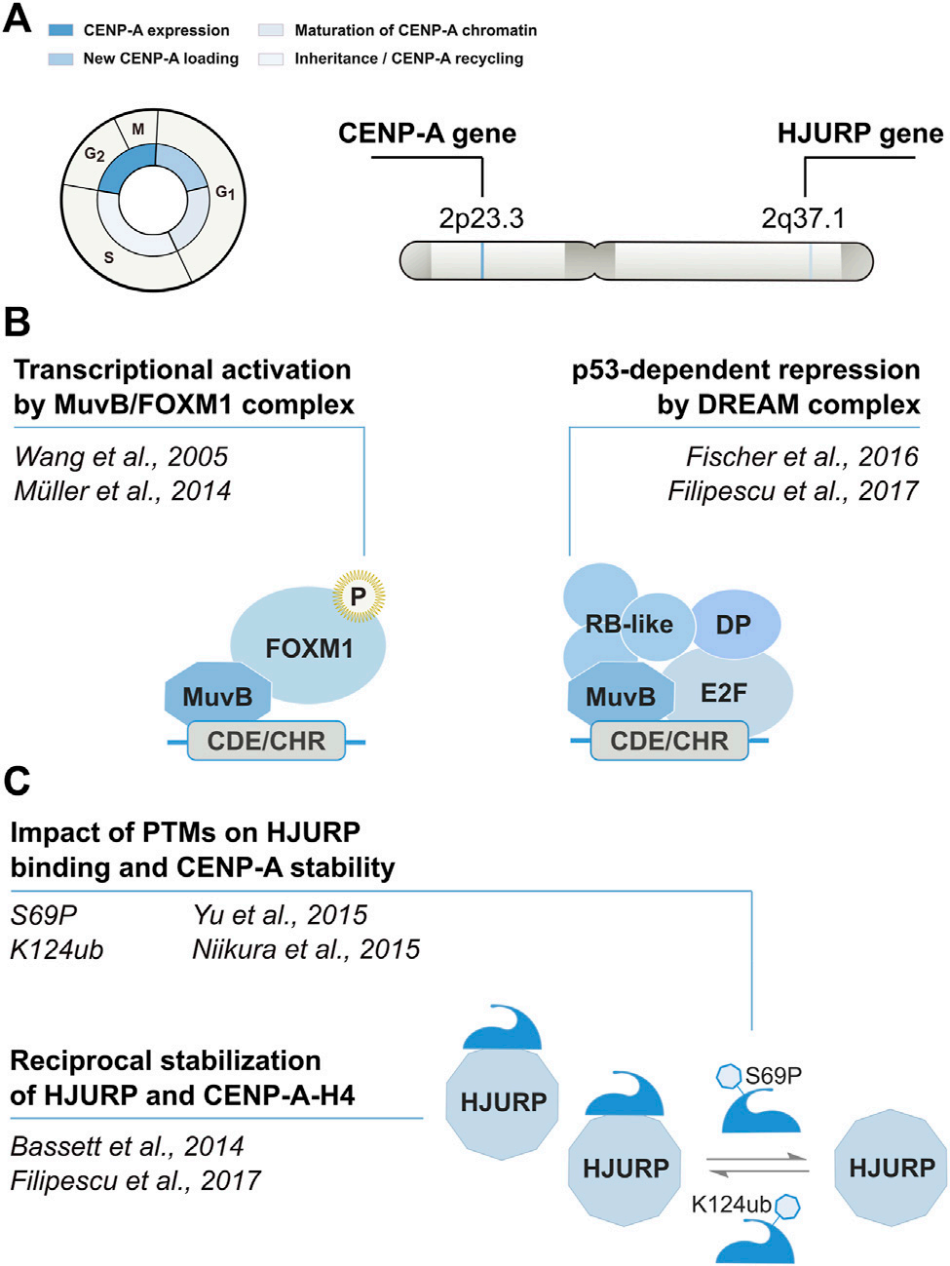
Cell cycle timing and regulation of CENP-A. Modified from Mendiratta et al., 2019 *.* **(A)** CENP-A and its chaperone HJURP are regulated in a cell cycle–dependent manner. **(B)** Transcription is regulated in cis by the CHR/CDE motif in their promoter region. The recruitment of the DREAM complex at the CHR is thought to promote transcriptional repression during G1. At the beginning of S phase, the MuvB core of the DREAM complex remains bound to the CHR/CDE while other components dissociate and are replaced by B-MYB. The B-MYB-MuvB (MMB) complex recruits FOXM1 in late S phase. B-MYB is degraded upon phosphorylation, whereas the progressive phosphorylation of FOXM1 leads to its activation in G2/M. **(C)** CENP-A and HJURP mutually stabilize each other at the protein level. The reciprocal stabilization is affected by posttranslational modifications of CENP-A.

CENP-A is encoded by a single multi-exon gene located outside of the histone gene clusters (Figure 1). The CENP-A gene (CENPA) undergoes conventional pre-mRNA processing *via* splicing and polyadenylation. Expression levels of CENP-A and its chaperone HJURP are cell cycle regulated, with a peak of expression in G2/M (Shelby et al., 1997) (Figure 1A). CENPA and HJURP transcription involves a CDE/CHR motif in their promoters recognized by the transcriptional regulator FOXM1 (Wang et al., 2005; Chen et al., 2013) and by the DREAM complex (Fischer et al., 2016). The DREAM complex is a transcriptional regulator binding to CDE/CHR sites to mediate indirect p53-p21-dependent repression, which can switch to an activator notably by recruitment of FOXM1 (reviewed in (Engeland, 2018)). These regulatory mechanisms provide means to keep these genes under a p53-dependent transcriptional repression (Filipescu et al., 2017) (Figure 1B). CENP-A expression levels vary depending on the tissue, in a range up to 50-fold changes, with the highest CENP-A expression scores found in lymphoid, skin and gastrointestinal tissues according to the Human Protein Atlas (Uhlén et al., 2015). In addition to transcriptional regulation, CENP-A amounts are also controlled at the protein level and CENP-A and its chaperone HJURP mutually stabilize each other (Bassett et al., 2012; Filipescu et al., 2017). Their reciprocal stabilization is affected by posttranslational modifications on the N-terminal tail of CENP-A, which can further regulate the timing of deposition. CENP-A S69 phosphorylation prevents interaction with HJURP and premature loading, while K124 ubiquitylation favors HJURP binding and contributes to their stabilization (Figure 1C). Thus, the overexpression of either CENP-A or HJURP leads to an increase in the levels of their binding partner (Filipescu et al., 2017). Conversely, HJURP loss results in CENP-A depletion, and CENP-A knockdown leads to the proteasome-mediated degradation of HJURP. Specific enrichment of CENP-A at the centromere is achieved in telophase/early G1 by HJURP (Dunleavy et al., 2009; Foltz et al., 2009), which is recruited at the centromere during this critical time window by the Mis18 complex [composed of two subunits, Mis18α and Mis18β, and the Mis18-binding protein 1 (Mis18BP1)] (Jansen et al., 2007; Dunleavy et al., 2009; Foltz et al., 2009; Zasadzińska et al., 2013; Nardi et al., 2016). Specifically, during M phase, CDK1 phosphorylates CENP-C, which stabilizes its interaction with CENP-A nucleosomes (Watanabe et al., 2019; Ariyoshi et al., 2021). CENP-A-bound CENP-C can recruit Mis18 to centromeres (Moree et al., 2011; Dambacher et al., 2012; McKinley and Cheeseman, 2014). Following this step, during anaphase, reduced CDK1/2 activity results in the dephosphorylation of HJURP, which in turn promotes its binding to Mis18 (Wang et al., 2014; Stankovic et al., 2017). Finally, in the second part of the G1 phase, the RSF (remodeling and spacing factor) complex (Perpelescu et al., 2009) and MgcRacGap (a Rho family GTPase activating protein) (Lagana et al., 2010) interact transiently with centromeres via HsKNL2, a G1-specific CENP-A licensing factor, to stabilize newly deposited CENP-A nucleosomes and generate mature centromeric chromatin. When the cell transitions to S-phase, CDK1/2 activity levels increase, Mis18BP1 is phosphorylated, released from centromeric chromatin, and CENP-A deposition is inhibited (Silva et al., 2012; McKinley and Cheeseman, 2014; Pan et al., 2017; Spiller et al., 2017). Thus, under normal conditions, a combination of mechanisms, some of which exploit the partnership between CENP-A and its dedicated chaperone HJURP, ensure that CENP-A levels and deposition are restricted to the centromere to promote faithful chromosome segregation.

## CENP-A Levels Increase in Cancer

**FIGURE 2:**
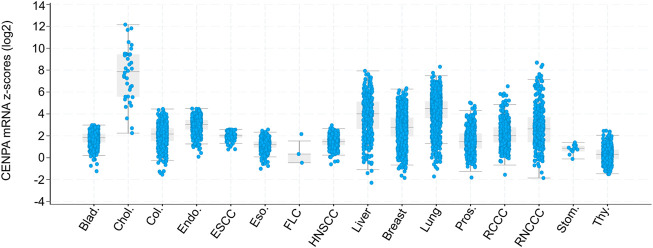
Box and dot plots indicating CENPA expression in patient tumors relative to normal samples (z-score, log2 RNA SeqV2 RSEM). Modified from Jeffery et al., 2021 *.* Box and dot plots indicating CENPA mRNA expression levels in patient tumors relative to normal samples (z-score, log2 RNA SeqV2 RSEM) from cBioPortal (TCGA PanCancer Atlas studies) (Cerami et al., 2012; Gao et al., 2013). The plot was generated using the cBioPortal web interface. X-axis indicates Cancer Type: Bladder Urothelial Carcinoma (Blad.), Cholangiocarcinoma (Chol.), Colorectal Adenocarcinoma (Col.), Endometrial Carcinoma (Endo.), Esophageal Squamous Cell Carcinoma (ESCC), Esophagogastric Adenocarcinoma (Eso.), Fibrolamellar Carcinoma (FLC), Head and Neck Squamous Cell Carcinoma (HNSCC), Hepatocellular Carcinoma (Liver), Invasive Breast Carcinoma (Breast), Non-Small Cell Lung Cancer (Lung), Prostate Adenocarcinoma (Pros.), Renal Clear Cell Carcinoma (RCCC), Renal Non-Clear Cell Carcinoma (RNCCC), Undifferentiated Stomach Adenocarcinoma (Stom.), Well-Differentiated Thyroid Carcinoma (Thy.). TCGA: The Cancer Genome Atlas.

Interestingly, by comparison with healthy tissues, elevated CENP-A expression levels are documented in many cancer types in humans, including breast (Ma et al., 2003; McGovern et al., 2012), colon (Tomonaga et al., 2003), liver (Li et al., 2011), lung (Wu et al., 2012), ovarian (Qiu et al., 2013), bone (Gu et al., 2014), gastric (Xu et al., 2020), and prostate (Saha et al., 2020). Indeed, the analysis of matched normal-tumor transcriptomes from 101 datasets demonstrated that the overexpression of CENP-A is a common feature of more than 44% of human cancers (Lacoste et al., 2014). Additionally, Oncomine analysis of 51 studies showed that CENP-A expression is significantly upregulated in 20 types of solid tumors compared with healthy tissues (Sun et al., 2016). Among these studies, the most common cancer types showing CENP-A overexpression were breast, central nervous system, ovary, and lung cancers. By contrast, down-regulation of CENP-A expression has never been observed in cancer tissues. More recently, analysis using TCGA PanCancer Atlas confirmed the global increase in CENP-A levels in patient tumors relative to healthy tissues, which reaches over 1000-fold in the case of cholangiocarcinoma (Jeffery et al., 2021) (Figure 2). In line with their co-regulation (see above), the unusual level of CENP-A often occurs in parallel with upregulation of its chaperone HJURP (Dunleavy et al., 2009; Jeffery et al., 2021). Thus, on average CENP-A mRNA levels are significantly and positively correlated with those of HJURP in several cancer types as assessed based on gene expression profiling interactive analysis (Zhang et al., 2016).

The negative regulation of CENPA and HJURP transcription under a p53 control (Fischer et al., 2016) is aligned with observations in mouse tumor cell line models where TP53 loss-of-function mutations lead to high CENP-A and HJURP levels (Filipescu et al., 2017). However, according to TCGA PanCancer Atlas studies visualized on CBioPortal, CENP-A overexpression can be observed in many tumors displaying TP53 loss of function mutation or wild-type (WT) TP53 (Cerami et al., 2012). Notably, early studies did not consider the p53 status of the tumors when assessing CENP-A levels. As a result, two distinct situations (p53-WT or p53-defective) were combined in the data concerning CENP-A levels and its link with cancer, thereby masking potentially important distinctions. Thus, beside the direct loss of p53 function, other mechanisms underly aberrant expression levels of CENP-A in cancer. Genetic analysis indicated that the elevated CENP-A expression was not due to alterations in the sequence or an increased copy number of the CENPA gene (Sun et al., 2016) and other factors, apart from a direct impairment in p53, deserve to be explored to explain CENP-A overexpression in human cancers. Several hypotheses can be considered. First, the tumor suppressor protein pRb (retinoblastoma protein), whose inactivation leads to CENP-A upregulation (Amato et al., 2009), is frequently mutated or disabled in cancer [reviewed in (Hickman et al., 2002)]. Secondly, CENP-A and HJURP overexpression could be the result of a deregulation of FOXM1, a known oncogenic transcription factor and activator of CENPA and HJURP transcription (Wang et al., 2005; Thiru et al., 2014). Indeed, the upregulation of CENP-A strongly correlates with the expression of FOXM1 across many cancers and FOXM1 displays at least 100-fold relative enrichment at the CENPA promoter in cancer tissues compared with normal tissues (Thiru et al., 2014). Finally, E2F8 and E2F2 are also potential transcriptional regulators of CENPA with critical roles in tumorigenesis (Sun et al., 2016). Importantly, whichever underlying mechanism leads to CENP-A upregulation, an excess of this particular histone H3 variant leads to an unbalanced situation in histones with effects onto chromatin organization as we will now discuss.

## CENP-A Overexpression Leads to Mislocalization and Heterotypic Nucleosomes

**FIGURE 3:**
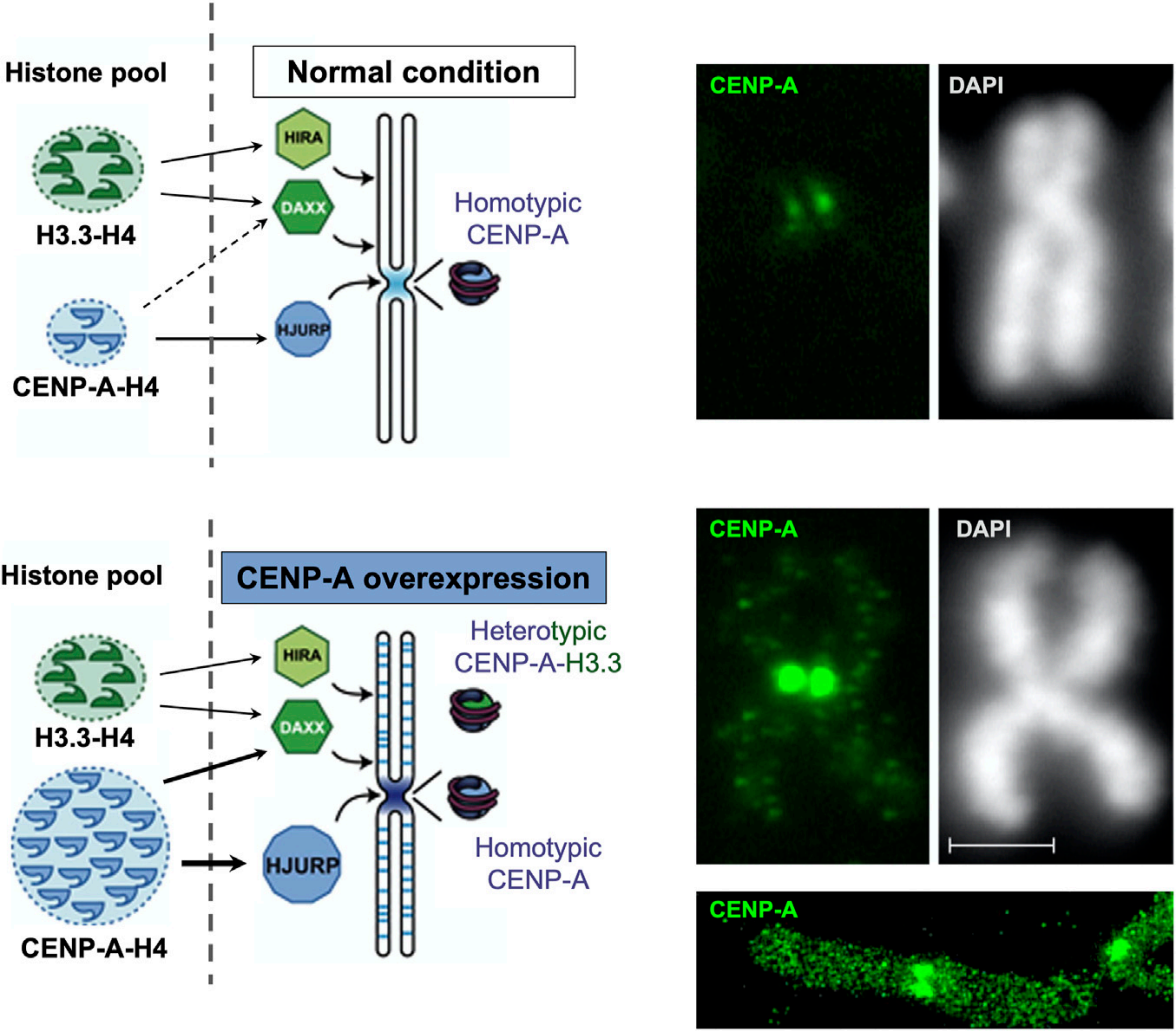
CENP-A overexpression leads to a mislocalization to chromosome arms with formation of heterotypic nucleosomes. Modified from Lacoste et al., 2014 *.* Left, scheme: in the normal situation (top), CENP-A is deposited specifically at the centromere by its dedicated chaperone HJURP and forms homotypic particles. Upon overexpression (bottom), CENP-A get mislocalized in the chromosome arms in a manner that depends on a H3.3 chaperone, DAXX. In these loci, the CENP-A particle is heterotypic. Right, immunofluorescence images for CENP-A detection on a mitotic chromosome compare normal (top) and CENP-A overexpressing (bottom) HeLa cells along with a DAPI staining. Scale bar = 2 μM. Below, a super-resolution image (courtesy of Tristan Piolot and Nicolas Lacoste) further illustrates the mislocalized CENP-A in chromosome arms.

The localization of CENP-A at the centromere is normally controlled by HJURP. Thus, consistently, when HJURP is overexpressed, the increased levels of CENP-A that ensue result in an exclusive accumulation at the centromere, without ectopic localization (Filipescu et al., 2017). Strikingly though, exogenous overexpression or abnormal upregulation of endogenous CENP-A leads to its mislocalization at non-centromeric regions (Van Hooser et al., 2001; Gascoigne et al., 2011; Lacoste et al., 2014; Athwal et al., 2015) (Figure 3). This is observed for overexpression of CENP-A either constitutive (Lacoste et al., 2014; Athwal et al., 2015; Shrestha et al., 2017, 2021) or inducible (Van Hooser et al., 2001; Gascoigne et al., 2011; Shrestha et al., 2017, 2021; Jeffery et al., 2021). Interestingly, the ectopic incorporation of CENP-A is abrogated when both CENP-A and HJURP are overexpressed in a coordinated fashion, as found following p53 inactivation by the oncogene E1A (adenovirus oncoprotein) and/or HRas-V12 (Ras oncogenic mutant). In this case, while CENP-A levels increase, its localization remains exclusive to the centromere (Filipescu et al., 2017). It is tempting, based on these findings, to propose that it could be when CENP-A overexpression exceeds the one of HJURP, that its mislocalization occurs (Lacoste et al., 2014; Athwal et al., 2015; Jeffery et al., 2021). This interdependence thus illustrates the functional importance of CENP-A and HJURP partnership to restrict CENP-A localization at the centromere. In CENP-A overexpressing cells, when HJURP capacity to chaperone CENP-A gets saturated, the excess of CENP-A tends to associate with other histone H3 chaperones. Under these conditions, while HJURP still ensures a deposition of CENP-A at the centromere, the excess pool of CENP-A gets ectopically deposited within chromosome arms through a DAXX-dependent histone deposition pathway (Lacoste et al., 2014). Thus, in this case the DAXX-ATRX complex, normally responsible for depositing H3.3 at other chromatin sites [reviewed in (Ray-Gallet and Almouzni, 2021)], is diverted to compensate for a saturation of HJURP. Other H3 chaperones like the HIRA complex have also been proposed to act as a means to compensate for CENP-A overexpression (Nye et al., 2018). The strict selectivity of histone-chaperone partners is thus weakened with unbalanced levels of CENP-A. Thus, reestablishing a correct balance between levels of HJURP and CENP-A can preclude ectopic assembly of CENP-A by H3.3 chaperones (Nye et al., 2018).

In addition to mislocalization, the excess of CENP-A which is ectopically incorporated show distinct properties in the corresponding nucleosomal core particles. Whereas at centromeric loci, homotypic particles with two CENP-A-H4 dimers form as (CENP-A-H4)_2_ tetramers, at ectopic loci, heterotypic nucleosomes combining both CENP-A and H3 variant are observed (CENP-A-H4,H3.3-H4) (Lacoste et al., 2014). Accumulation of ectopic CENP-A particles is mainly observed at sites of high histone turnover (Lacoste et al., 2014; Mahlke and Nechemia-Arbely, 2020) such as active promoters and enhancers (Lacoste et al., 2014), as well as subtelomeric and pericentromeric regions (Athwal et al., 2015; Nye et al., 2018). Moreover, ectopic CENP-A has also be found over CTCF binding sites, precluding proper CTCF localization at these sites (Lacoste et al., 2014). Given the importance of CTCF in 3D genome organization, how these CTCF displacements may impact chromatin architecture in the nucleus should be explored.

## Impact of CENP-A Overexpression on Kinetochore and Chromosome Segregation

Assembling all components of the kinetochore is an important property of CENP-A to ensure a proper centromere function (Liu et al., 2006; Fachinetti et al., 2013). A major issue is thus to determine what happens at sites where CENP-A is mislocalized when overexpressed, since, in theory, this could potentially lead to the formation of neocentromeres competing with the original centromere. In fact, in human cells, ectopic CENP-A recruits only a subset of kinetochore proteins, namely CENP-C, CENP-N and the Mis18 complex (Van Hooser et al., 2001; Gascoigne et al., 2011; Lacoste et al., 2014), whereas the NDC80 complex has not been found. Therefore, overexpressing CENP-A is not sufficient to form stable neocentromeres in human cells. Since active neocentromere formation only occurs following inactivation of an endogenous centromere (Marshall et al., 2008), as long as the endogenous centromere is functional it may prevent the possibility for neocentromere to form. Indeed, following inactivation of endogenous centromere, ectopic CENP-A is capable of seeding stable neocentromere formation (Shang et al., 2013). Recently, Murillo-Pineda et al., using a CRISPR-Cas9 approach to remove the initial centromeric region from a copy of chromosome 4 in human retinal pigment epithelium cells, observed the spontaneous formation of a neocentromere correlating with CENP-A seeding and eviction of H3K9me3 (Carty and Dunleavy, 2021; Murillo-Pineda et al., 2021). These data support an important role of non-centromeric CENP-A deposition in neocentromere formation. However, conversely, the ectopic CENP-A traps a series of kinetochore components such as CENP-C, CENP-T and Nuf2 (a member of the Ndc80 complex) away from native centromeres, resulting in their weakening (Shrestha et al., 2017, 2021). Thus, these human cells exhibit mitotic defects such as lagging chromosomes, micronuclei formation, abnormal mitotic exit, and overall aneuploidy with karyotypic heterogeneity (Amato et al., 2009; Athwal et al., 2015; Shrestha et al., 2017, 2021). Of note, many of these chromosome segregation defects are suppressed when DAXX is depleted from CENP-A overexpressing cells (Shrestha et al., 2017, 2021). Altogether, these data indicate that ectopic incorporation of CENP-A into chromosome arms due to CENP-A overexpression represents a major source of mitotic stress that results in chromosomal instability (CIN) and aneuploidy. This in turn can lead to a large heterogeneity in the derived cell populations.

## CENP-A Overexpression and Cell Fate Reprogramming

**FIGURE 4:**
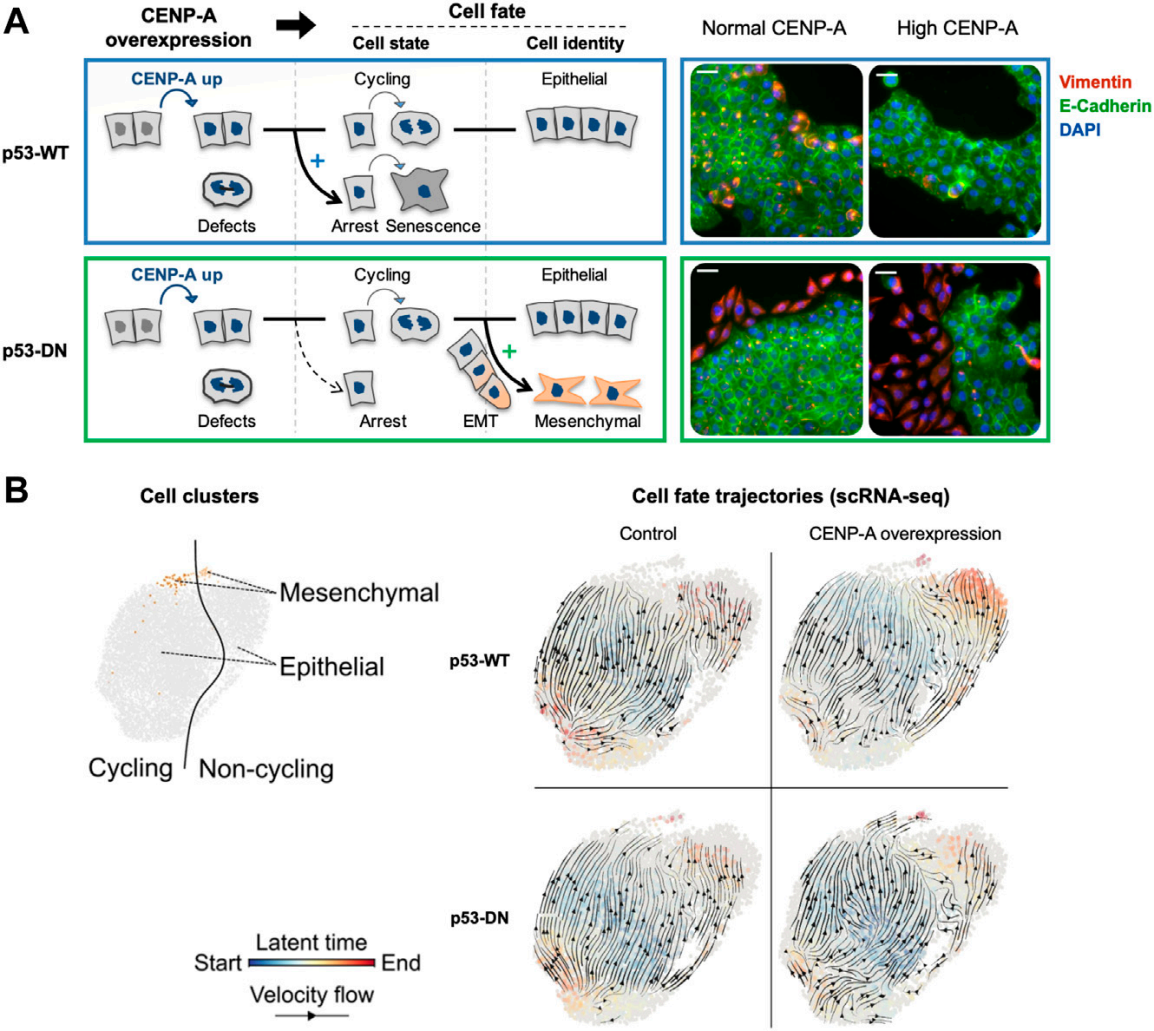
CENP-A overexpression promotes distinct cell fates depending on p53 status. Modified from Jeffery et al., 2021 *.* **(A)** Scheme summarizing the consequences of CENP-A overexpression (in blue) on cell state and identity, in wild-type p53 (p53-WT, top panel blue) and p53-defective cells (p53-DN, dominant negative, bottom panel green). IF images show representative max intensity projections after 34 days with or without continuous CENP-A overexpression. DAPI (blue), E-cadherin (green, epithelial marker), and vimentin (red, mesenchymal marker). Scale bars = 40 µM. **(B)** Cell fate trajectories inferred by RNA velocity analysis of scRNA-seq samples. RNA velocities (black arrows) were calculated for each condition by stochastic modeling in individual samples. Each cell is colored by the estimated latent time from initial (blue, start) to terminal states (red, end).

Since chromatin states have been associated with transcription regulation and cell fate, it is important to understand how CENP-A overexpression and its impact on chromatin could impact transcription and cell fate. Cancer cells generally activate transcriptional programs that ensure an upregulation of both core kinetochore components and genes required for cell cycle progression and DNA replication (Thiru et al., 2014). Analysis of gene expression dynamics associated with CENP-A overexpression showed major changes in most genes involved in cell-cycle progression, apoptosis, DNA repair and RNA metabolism (Li et al., 2011; Sun et al., 2016; Jeffery et al., 2021). Interestingly, hotspots of ectopic CENP-A genomic localization had documented transcriptional start sites of genes, like CDC25C (Saha et al., 2020) and the oncogene MYC (Athwal et al., 2015). Furthermore, CENP-A mislocalization to the MYCN locus showed strong correlation with the amplification and overexpression of MYC (Nye et al., 2018). In addition, as mentioned above, aberrant CENP-A nucleosomes can occlude CTCF binding sites (Lacoste et al., 2014). This is particularly relevant when considering that the binding of CTCF is frequently altered in cancer and associated with tumorigenicity (Kemp et al., 2014; Katainen et al., 2015; Marshall et al., 2017; Aitken et al., 2018). However, while CENP-A overexpression could push/facilitate a transition for cells to progress towards cancer, alone, its overexpression does not affect proliferation rate and is not sufficient for tumorigenic transformation, even with a loss of p53 function (Filipescu et al., 2017). Thus, the impact of CENP-A overexpression is likely to operate in combination with other events occurring during oncogenesis and be dependent on the cellular context. A first context to consider is the difference in p53 status, and recent work examining various cellular contexts underlined the importance of the p53 status in the transcriptional reprogramming induced by CENP-A. This was achieved by comparing side by side, p53-WT cells and a counterpart where p53 status is altered with a dominant negative p53 (p53-DN). In the p53-WT cells, CENP-A upregulation could promote cell cycle exit and senescence whereas, in p53-defective cells, the shift from cycling to non-cycling cells was reduced and CENP-A overexpression lead to the emergence of a subpopulation of cells with downregulated epithelial genes but upregulated mesenchymal genes (Jeffery et al., 2021) (Figure 4). Thus, CENP-A, in a p53 dependent manner, can impact cell fate in two major ways: first with respect to cell cycle by evading from a senescence program, and second by promoting a cellular reprogramming favoring a change of cell identity, namely the epithelial-mesenchymal transition (EMT). EMT is an essential process during mammalian embryonic development and in wound healing [reviewed in (Yang et al., 2020)]. However, tumor cells hijack this process to migrate more efficiently and invade the underlying mesenchyme (Ye et al., 2017; Yang et al., 2020). Thus, in cancer, EMT has been associated with invasion, metastasis, and resistance to therapy [reviewed in (Yang et al., 2020)]. Similarly, high CENP-A levels correlate strongly with tumor aggressiveness, increased invasiveness and metastasis in cancer patient data (Ma et al., 2003; McGovern et al., 2012; Gu et al., 2014; Sun et al., 2016; Zhang et al., 2016; Saha et al., 2020). Of note, a recent study also linked CENP-A overexpression to invasion and metastasis via activation of the β-catenin pathway in clear cell renal cell carcinoma (Wang et al., 2021). In cell culture, CENP-A overexpression has been associated with reduced expression of cell adhesion genes and higher cell invasion (Shrestha et al., 2021). Thus, the induction of EMT could explain the invasiveness and metastasis capacity of particular tumors overexpressing CENP-A. It will be important in the future to elucidate the exact mechanisms and pathways underlying EMT activation promoted by CENP-A. Indeed, CENP-A overexpression alone is not sufficient to induce EMT in any p53-defective cell line (Jeffery et al., 2021), only certain cells can undergo these transitions. Whether the effect on gene expression requires a certain threshold of CENP-A overexpression or is cell-type specific remains to be established. An attractive hypothesis to test is that CENP-A overexpression would function in the context of cells already in transition (e.g., cells in tumorigenic progression). Indeed, its targeting in active regions could particularly alter gene expression where both CTCF and the presence or absence of specific histone variants play a critical role (Goldberg et al., 2010; Szenker et al., 2012). The current emerging view is that CENP-A would help to maintain/stabilize the population of cells transitioning. How this is actually operating mechanistically remains to be determined. It is tempting to consider as well how CENP-A changes could impact the organization of the genome within the nucleus to possibly contribute to changes in gene expression and function, thus affecting cell fate (Krijger and de Laat, 2013; Bonev and Cavalli, 2016; Fritz et al., 2019). However, this latter aspect has not yet been examined.

## CENP-A Nuclear Distribution in Health and Cancer

**FIGURE 5:**
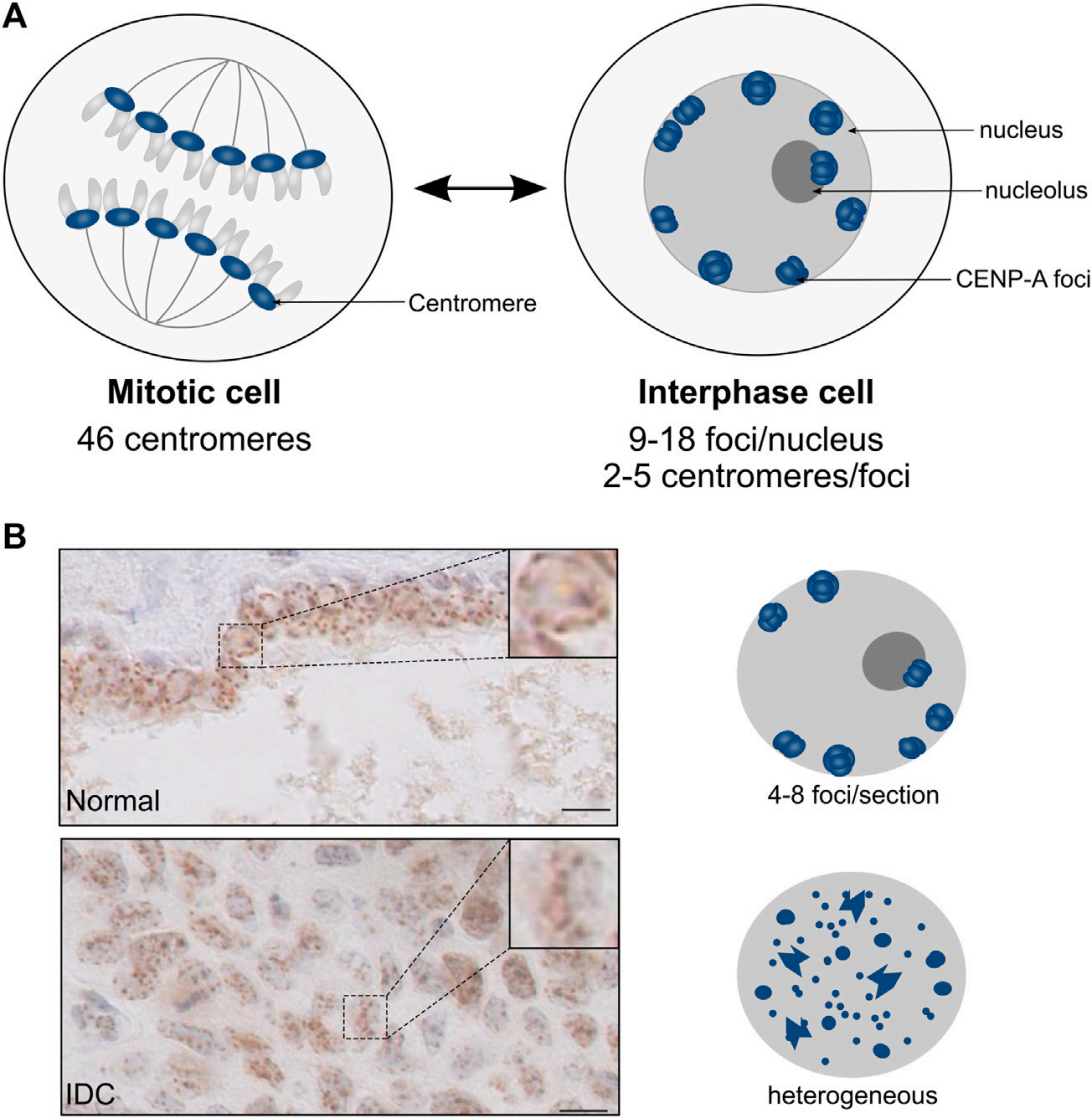
CENP-A localization patterns in normal tissues and neoplastic lesions. Modified from Verrelle et al., 2021. **(A)** Schematic representation of nuclei from mitosis (left) and interphase (right) cells. In interphase cells, the clustering of centromeric CENP-A-enriched regions (blue) gives rise to CENP-A foci at the periphery of the nucleus and, in fewer cases, of the nucleolus. **(B)** CENP-A localization patterns visualized by IHC in breast tissues. Top: Normal duct; Bottom: Invasive Ductal Carcinoma (IDC). The boxed area shows the magnification of a representative nucleus. Scale bar = 10 µM. On the right is shown a schematic representation of the CENP-A localization pattern, with CENP-A foci (blue) within the nucleus (grey).

In the interphase nucleus, chromosomes are non-randomly arranged within the nuclear space. They are structurally and functionally organized into territories, and this nuclear organization is generally altered in cancer (Lever and Sheer, 2010; Zaidi et al., 2018; Mai, 2019). Notably, centromeres occupy particular subnuclear domains in interphase nuclei [review in (Muller et al., 2019)]. Within this organization, the DNA-binding protein CENP-B favors loops between DNA repeats (Chardon et al., 2022). These subnuclear localization of centromeres provide information that can be followed according to the cell fate and differentiation (Jansz and Torres-Padilla, 2019; Schlesinger and Meshorer, 2019; Feodorova et al., 2020). To date, however, much of our knowledge comes from data collected in mammalian cultured cells. In these proliferating cells, CENP-A marks the centromere of the mitotic chromosomes and, during interphase, these marks are detected as numerous foci dispersed throughout the nucleus (Dunleavy et al., 2009; Lacoste et al., 2014; Verrelle et al., 2021). Importantly, these cultured cells derived from tumors provide a CENP-A localization in interphase that cannot necessarily reflect the situation in normal cells. Indeed, in normal human primary lymphocytes, the visualization of centromeres both by FISH (Skalníková et al., 2000; Weierich et al., 2003) and CENP-A staining (Weimer et al., 1992; Alcobia et al., 2000) provided very defined nuclear patterns quite distinct from the tumoral cells. Furthermore, in the cell nuclei of human normal solid tissues, CENP-A is detected in 9–18 foci corresponding to the clustering of the centromeres from 2 to 5 distinct chromosomes. These foci, homogenous in size and shape, localize equidistantly at the nuclear periphery and, in fewer cases, at the periphery of the nucleolus (Figure 5) (Verrelle et al., 2021). This very distinct organization represents a general feature shared by distinct cell types originating from various progenitors and along various different differentiation pathways irrespective of the fact that cells could be circulating or in tissues. Thus, in line with the hypothesis that centromeric regions are robust and stable chromosomal loci (Krijger and de Laat, 2013), CENP-A labelling as foci at the nuclear periphery reflects a fundamental feature of centromeres organization and distribution in healthy cells. By contrast, in human tumor tissues, this discrete CENP-A subnuclear localization is drastically altered and, for invasive neoplastic lesions, similar to that detected in the cultured cell lines of tumoral origin (Verrelle et al., 2021). Interestingly, CENP-A subnuclear localization in breast lesions reveals distinct information. In healthy tissues, the normal reference pattern compares to benign lesions (Verrelle et al., 2021). By contrast, in neoplastic lesions, CENP-A foci localization in a regular distribution at the nuclear periphery is disrupted and clustering is altered or lost (smaller and many more foci) (Figure 5). Furthermore, the loss of CENP-A foci localization at the nuclear periphery with a high heterogeneity in terms of number, size, and shape within the nuclear volume can readily discriminate invasive from non-invasive neoplastic lesions. These major global change of centromere and nuclear organization in cancer reported recently (Verrelle et al., 2021) have not yet been related to ectopic localization of CENP-A in cancer (Lacoste et al., 2014). How these changes during oncogenic transformation and progression could possibly relate thus remains to be assessed. Notably, tumor grade is reported to combine major perturbations that can lead to genome chaos (Liu et al., 2014). This destabilizing context might reach a threshold above which normal centromeric loci and CENP-A nuclear organization cannot be maintained. Whether the change of CENP-A subnuclear localization in tumor cells/tissues is a cause or a consequence of oncogenesis remains an open question (Maison et al., 2010; Finn and Misteli, 2019; Muller et al., 2019). Given that the mechanisms involved in the clustering of centromeres and their link with the control of nuclear organization are yet to be discovered, the mechanisms contributing to the maintenance loss of CENP-A nuclear organization during tumorigenesis represent an exciting avenue to explore.

## Clinical Interest for CENP-A in Cancer Treatment

Elevated CENP-A levels correlate with cancer progression (Ma et al., 2003; Li et al., 2011; Qiu et al., 2013; Sun et al., 2016) and poor patient outcome (Ma et al., 2003; Li et al., 2011; McGovern et al., 2012; Wu et al., 2012; Qiu et al., 2013; Gu et al., 2014; Sun et al., 2016; Zhang et al., 2016; Saha et al., 2020). This correlation could reflect the CENP-A-induced CIN that leads to an increase in chromosomal rearrangements and mutagenesis rate, which both increase the heterogeneity of the cell population, thereby promoting the selection of advantageous clones. An alternative explanation could relate to the essential role of CENP-A in mitotic progression (Dunleavy et al., 2009; Foltz et al., 2009; Fachinetti et al., 2013; Filipescu et al., 2017). Following malignant transformation, hyperproliferative cells require sufficient expression of CENP-A along with its chaperone HJURP to ensure a proper CENP-A deposition enabling accurate chromosome segregation (Filipescu et al., 2017). This has led to the hypothesis according which such cells become “addicted” to CENP-A (Filipescu et al., 2017). In line with this hypothesis, in cancer cells overexpressing CENP-A, its depletion suppresses cell growth both *in vitro* and *in vivo*, blocks cell-cycle progression, and promotes apoptosis (Li et al., 2011). Similarly, CENP-A partial depletion (Li et al., 2011; Wu et al., 2014) reduces proliferation, growth, migration, and invasiveness of cancer cells. Thus, CENP-A is required for tumor progression and its depletion may have anticancer effects which are likely mediated by genes involved in cell cycle control and apoptosis (Li et al., 2011; Wu et al., 2014). These two reports using cancer cell models show promising potential for targeting CENP-A as a cancer therapeutic, as an Achille’s heel in addicted cells.

While CENP-A is generally associated with tumor aggressiveness, the correlation of high CENP-A levels with clear therapeutic response is not simple. Indeed, while a reduced therapeutic response is reported when CENP-A is overexpressed in osteosarcoma (Gu et al., 2014), some reports in breast (McGovern et al., 2012; Sun et al., 2016; Zhang et al., 2016) and lung (Zhang et al., 2016) cancers indicate a better clinical outcome. These discrepancies might reflect different cancer types/sub-types, various population of cells, possible heterogeneity in the samples and the methods of analyzing the therapeutic response. Considering the importance of the cellular context upon CENP-A overexpression, the p53 status clearly represented an important parameter to consider specifically (see discussion above). Indeed, CENP-A overexpression impacts cellular response to genotoxic stress, increasing the survival of p53-defective cells in the presence of DNA damage induced by a topoisomerase inhibitor (Lacoste et al., 2014). In addition, CENP-A overexpression strongly increases sensitivity to X-ray irradiation of several p53-WT cell lines but not of p53-defective cell lines (Jeffery et al., 2021). Importantly, the inactivation of p53 is sufficient to counteract the CENP-A induced radio-sensitivity phenotype (Jeffery et al., 2021). Thus, CENP-A acts as a double-edged sword: its overexpression enhances radio-sensitivity in cells with wild type p53, however, p53-defective cells show a more tolerant state to X-ray irradiation. This exemplifies how CENP-A overexpression could lead to either sensitivity or resistance to treatment depending on the p53 status. These findings highlight the importance of integrating the p53 status when considering CENP-A for cancer treatment. Indeed, while tumors displaying high CENP-A levels in a wild-type p53 context would respond to radiotherapy, to treat tumors with defective p53 alternative options should be considered. These could include drugs that can recover an active p53 in combination with radiotherapy, and/or other DNA damaging agents. Importantly, a number of p53-targeted drugs are now being tested in clinical trials (Blandino and Di Agostino, 2018; Bykov et al., 2018), including direct reactivators of mutant p53 (e.g. PRIMA-1MET/APR-246) and drugs designed to return functionality to wild-type p53 by inhibiting its upstream regulators (e.g. MDM2/X inhibitors) (Sanz et al., 2019). Interestingly, despite the impact on transcription of genes associated with DNA repair, CENP-A overexpression did not affect the rate of DNA repair (Jeffery et al., 2021), suggesting that sensitization or resistance observed is not related to repair efficiency.

Whether CENP-A can serve as a biomarker to improve stratification of cancer patients is also a crucial question. CENP-A overexpression has already been used as a general biomarker of poor patient prognosis and as a predictive biomarker for chemotherapy (Ma et al., 2003; Sun et al., 2016; Zhang et al., 2016). It has been proposed as a biomarker for tumor aggressiveness in ovarian (Qiu et al., 2013), osteosarcoma (Gu et al., 2014), lung (Wu et al., 2012; Liu et al., 2018), and breast estrogen receptor positive (McGovern et al., 2012; Montes de Oca et al., 2015) cancers. Interestingly, in breast cancer, identifying high levels of the CENP-A chaperone, HJURP, has been proposed as a prognostic marker of poor patient outcome, distinguishing aggressive tumors within the luminal A subtype (Montes de Oca et al., 2015). In addition to the use of CENP-A level, measured as a total amount in a bulk population to serve as a biomarker, given the discrete pattern observed, its nuclear distribution should also be considered. Indeed, CENP-A nuclear localization pattern in patient samples allows to directly interrogate the chromatin architecture in the nucleus by following centromeres and in breast could reveal changes between healthy, non-neoplastic and neoplastic lesions (Verrelle et al., 2021). CENP-A subnuclear pattern could also reveal clear alterations in chromatin architecture in the nucleus associated with resistance to treatments. The application of this strategy in head and neck squamous cell carcinoma (HNSCC) enabled to reveal a nuclear CENP-A localization pattern assessed by immunohistochemistry, as a predictive marker of local disease control at 2 years by concurrent chemoradiation therapy (CCRT) with 96% accuracy (Verrelle et al., 2021). This distinct CENP-A pattern, termed “Pattern-C” provided a strong prognostic marker of overall survival advantage, outperforming all other tumor characteristics usually examined including proliferation, HPV status and tumor stage. Given the general nature of CENP-A subnuclear patterns across tissues, extending the use of this simple staining method to predict sensitivity to concurrent chemoradiation may prove of interest for other cancers treated with genotoxic agents.

## Concluding Remarks

CENP-A is key for centromere function, a chromosome region with a fundamental role in cell division. However, recent work has demonstrated that beyond its centromeric role in mitosis, CENP-A may be related to tumor status through an impact on chromatin organization in interphase. We have presented recent studies concerning the regulation of CENP-A, and how aberrant expression affects mammalian cells in the context of cancer. The role of CENP-A in cancer progression is multifaceted, affecting both genome integrity and transcriptional reprogramming. These changes facilitate cancer progression and the acquisition of properties promoting metastasis. Furthermore, CENP-A levels could contribute to guiding therapy choice, notably when taking p53 status into account. Interestingly, in addition, CENP-A subnuclear pattern visualized on tissue sections represents a biomarker of potential prognostic value. Future work with new technological advances enabling to follow at a single cell level an integrated analysis of proteins, transcripts and spatial distribution in a tissue should help to further characterize the molecular mechanisms underlying the connections between CENP-A and cancer.
